# Morphology and Histology of Lyonet's Gland of the Tropical Tasar Silkworm, *Antheraea mylitta*

**DOI:** 10.1673/031.012.12301

**Published:** 2012-10-26

**Authors:** Sudip Patra, Ravindra Nath Singh, Mohammad Raziuddin

**Affiliations:** ^1^ University Department of Zoology, Vinoba Bhave University, Hazaribag- 825301. Jharkhand, India; ^2^ Central Silk Board, CSB Complex, BTM Layout, Madiwala, Bangalore - 560068, India

**Keywords:** Daba TV ecorace, silk gland

## Abstract

The morphology and histology of Lyonet's gland in the second to fifth instar larvae of *Antheraea mylitta* Drury (Lepidoptera: Saturniidae) are described. Each of the paired silk glands of this silk worm were associated with a Lyonet's gland. The paired Lyonet's glands were located on the ventrolateral sides of the esophagus, close to the subesophageal ganglion. Whole mount and SEM observations revealed that each Lyonet's gland consisted of a rosette of glandular mass, and a short narrow tubular duct opening into the anterior part of the silk gland (ASG), close to the common excretory duct. In each instar, these glands were unequal in size. The glandular mass was innervated by fine nerves from the subesophageal ganglion, suggesting a neural control for the glandular activity. The glandular mass was made up of clustered long cells wrapped by a thin basal lamina, which was continuous over the non-secretory low columnar cells of the Lyonet's gland duct and ASG. The narrow bases of long cells of each glandular mass led into the lumen of the duct of the gland. Histochemical analysis of fully developed Lyonet's gland showed clustered lipid granules in the gland cells.

## Introduction

The Lyonet's gland, first described in 1760 by Lyonet in lepidopteran larvae (Machida 1965), is often referred to as “Filippi's gland” in the silkworm *Bombyx mori* L. (Lepidoptera: Bombycidae) (Waku and Sumimoto 1974; Akai 1984). This gland usually occurs close to the excretory duct of the silk gland, and communicates with it (Waku and Sumimoto 1974). It has been considered as an accessory gland of the silk gland (Waku and Sumimoto 1974; Sehnal and Akai 1990).

The function of Lyonet's gland is still uncertain (Victoriano and Gregorio 2004). Its role in the exchange of small molecules, such as water and ions (Waku and Sumimoto 1974), in the secretory process of cementing substance for the silk elements (Day and Waterhouse 1953; Wigglesworth 1972 ), and secretion of some lubricating substance that helps in the extrusion of silk from the silk glands (Glasgow 1936; Day and Waterhouse 1953) have been suggested.

*Antheraea mylitta* D. (Lepidoptera: Saturniidae) is the producer of commercial tasar silk in tropical India. A survey of literature reveals that no information is available on the Lyonet's glands in the larvae of this silk moth. The present work is an attempt to describe the morphology, histology, and histochemical properties of these glands.

## Materials and Methods

Second to fifth instar larvae of *A. mylitta* (Daba TV ecorace) were procured from the field during rearing periods from Tasar Pilot Project Centre, Salboni, Purulia (West Bengal). The Lyonet's glands were removed, and fixed in appropriate fixatives for whole mounts, histology and histochemical studies. The glands of five larvae of each second to fifth instars were measured using the micrometer. 6µ thick sections of the gland were stained with Hematoxylin and Eosin/Triple Mallory, Mercuric bromophenol blue, PAS reagents, and Sudan black-B. For scanning electron microscopy (SEM), the Lyonet's glands of fifth instar larvae were fixed in 2.5% glutaraldehyde in 0.1 M phosphate buffer (P^H^ 7.2 to 7.4) at 4° C for 2– 3 hours, and then post-fixed in 1% osmium tetroxide in a similar buffer for 2 hours. The post-fixed specimens were dehydrated through graded series of alcohol and acetone, critical point dried with liquid CO2, and gold coated in a sputter. Scanning of specimens was performed by field emission scanning electron microscope.

## Results

**Figure 1.**
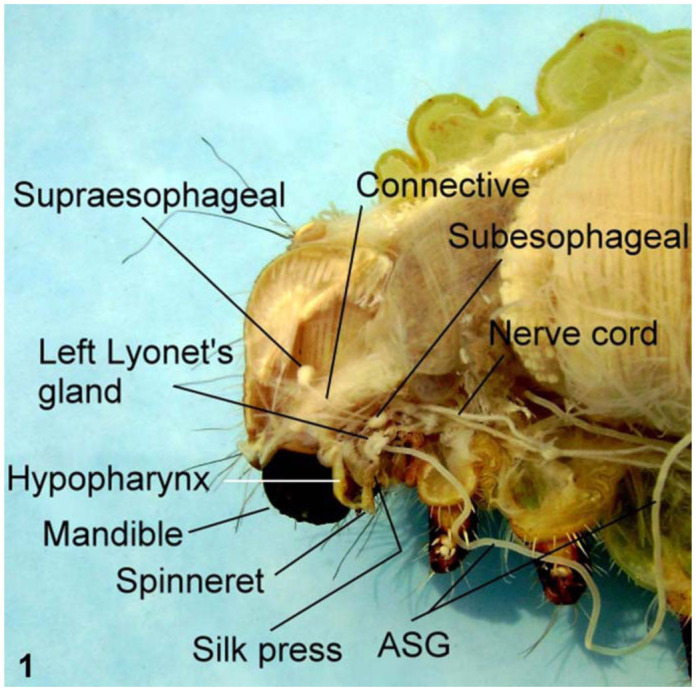
Dissected anterior region of fifth instar larva of *Antheraea mylitta* showing the location of Lyonet's gland close to the suboesophageal ganglion. High quality figures are available online.

**Figure 2.**
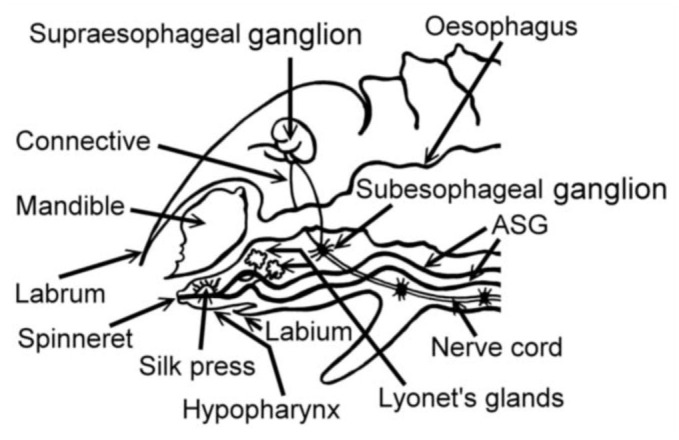
Location of Lyonet's glands in *Antheraea mylitta* larva (Diagrammatic). High quality figures are available online.

**Figure 3.**
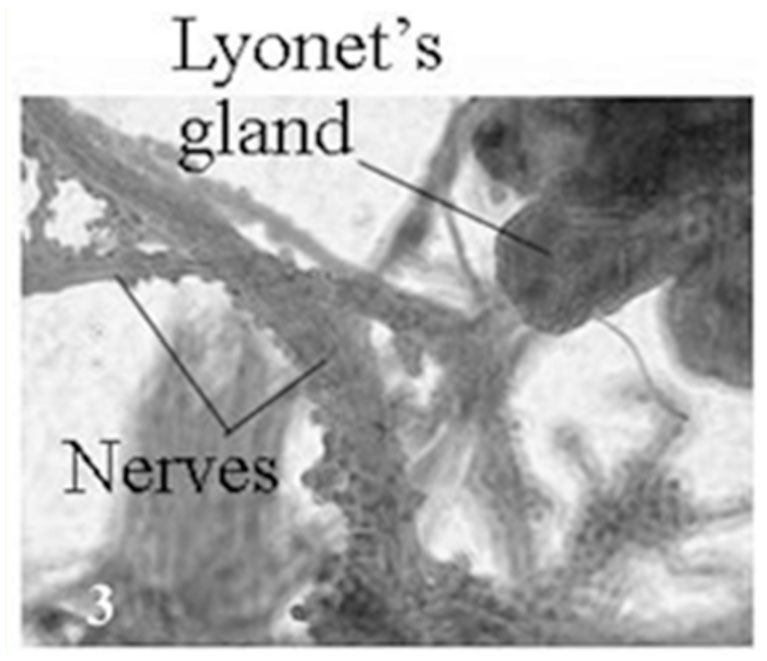
Lyonet's gland and associated nerves from the suboesophageal ganglion in *Antheraea mylitta* larva (w.m, × 1000). High quality figures are available online.

**Figure 4.**
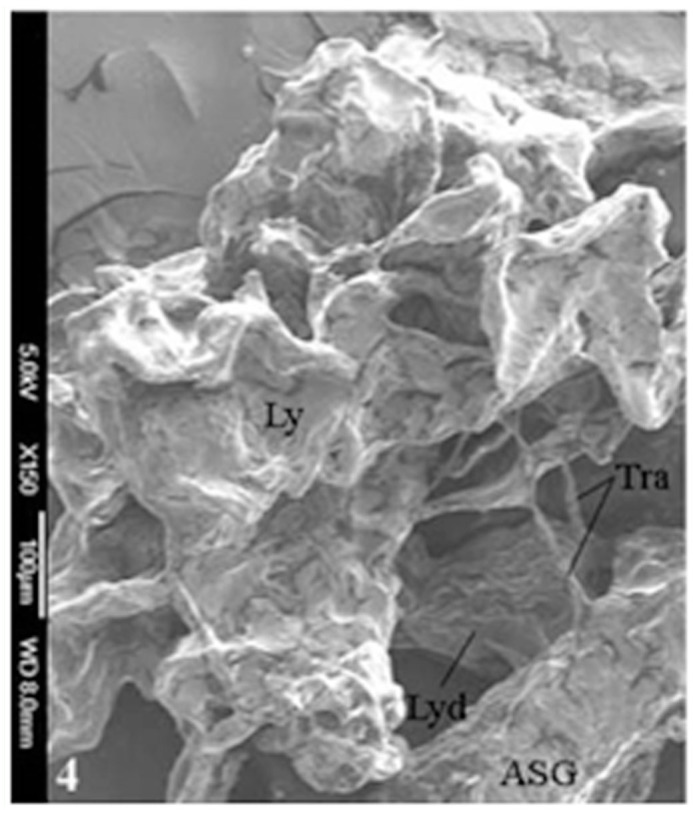
SEM image of Lyonet's gland surface in *Antheraea mylitta* larva. High quality figures are available online.

**Figure 5.**
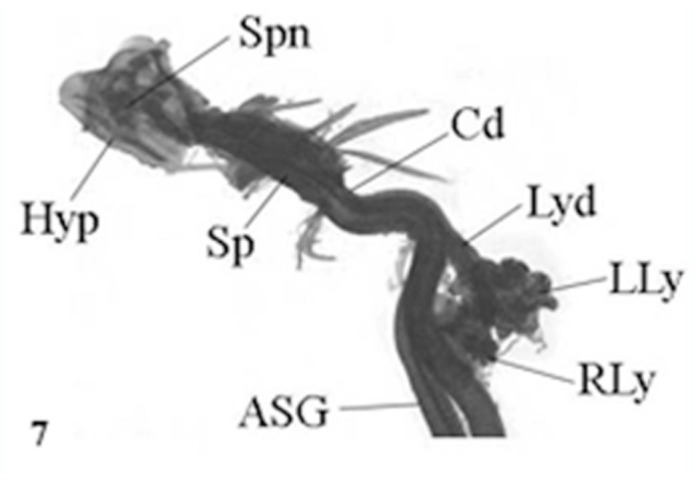
Lyonet's glands of fourth instar *Antheraea mylitta* larva (w.m. × 50). High quality figures are available online.

**Figure 6.**
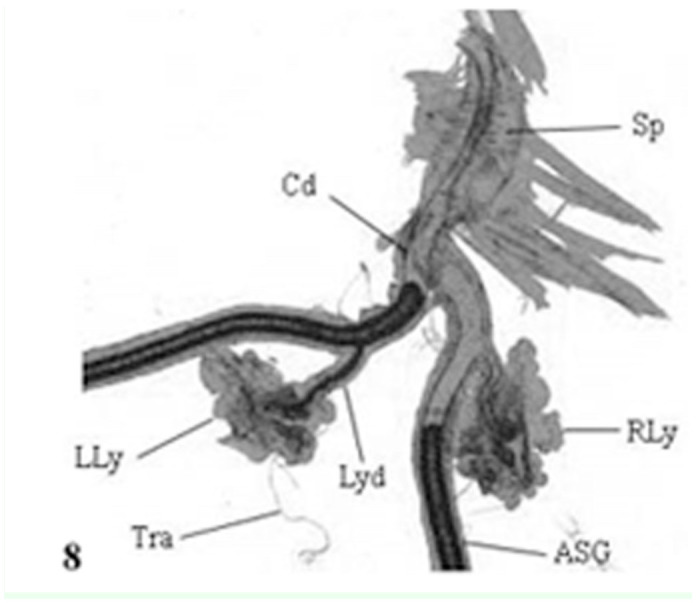
Lyonet's glands of fifth instar *Antheraea mylitta* larva (w.m. × 50). High quality figures are available online.

In *A. mylitta*, there was a pair of small, creamy white Lyonet's glands, each associated with the anterior parts of paired silk glands. These were located on the ventrolateral sides of the esophagus, close to the subesophageal ganglion (Figures 1, 2). Fine nerve fibers arising from the subesophageal ganglion innervated the Lyonet's gland, indicating a neural control for glandular function (Figure 3). Whole mount of the glands and SEM studies (Figure 4) revealed that each gland was made up of a rosette of glandular mass, and a narrow tubular duct that opens into the inner side of the anterior part of the silk gland (ASG), near the origin of common excretory ducts (Figures 4, 5, 6). These glands increased in size as the larval instar advanced. It was interesting to note that in the second to fourth instar larvae, the left Lyonet's gland was larger than the right gland, but in the fifth instar, the right Lyonet's gland was larger than the left one (Figures 5, 6). The morphometric records of the glands and their ducts from second to fifth instar larvae are presented in Table 1.

**Table 1.**
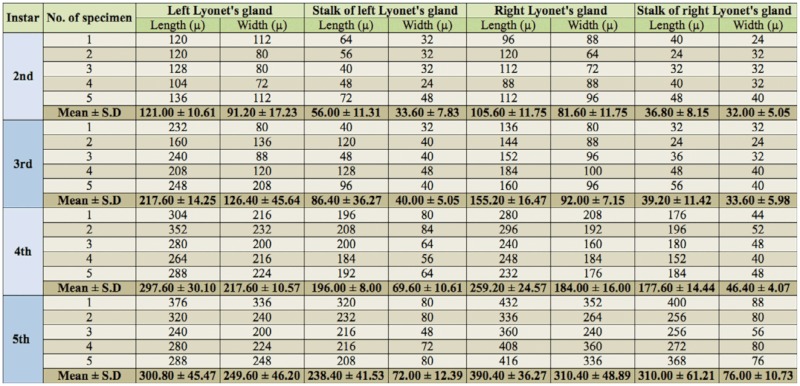
Measurement of Lyonet's glands and their ducts in five, second to fifth instar larvae of *Antheraea mylitta*.

**Figure 7.**
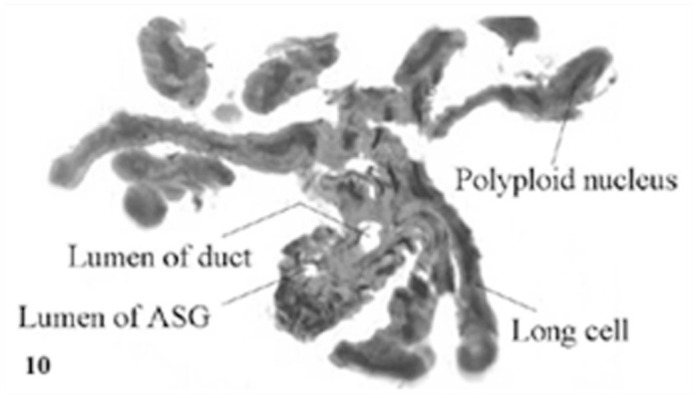
L. S. Lyonet's gland of third instar *Antheraea mylitta* larva showing cells and polyploid nuclei (× 450). High quality figures are available online.

**Figure 8.**
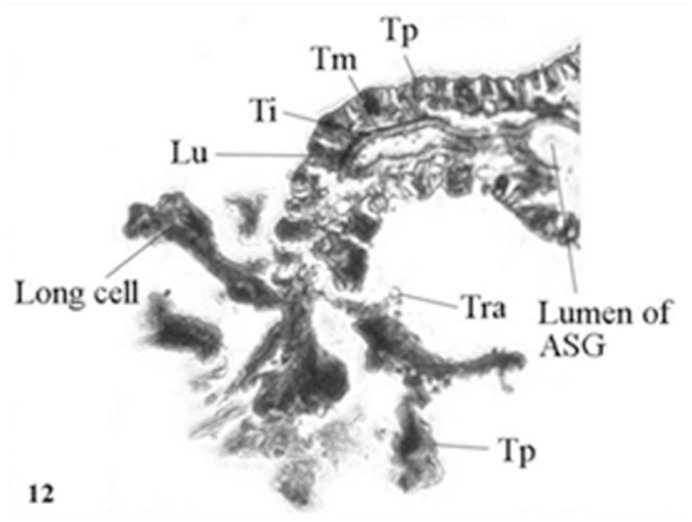
L. S. Lyonet's gland of fifth instar *Antheraea mylitta* larva, showing the long cells and continuity of its duct with the lumen of anterior silk gland (× 50). High quality figures are available online.

**Figure 9.**
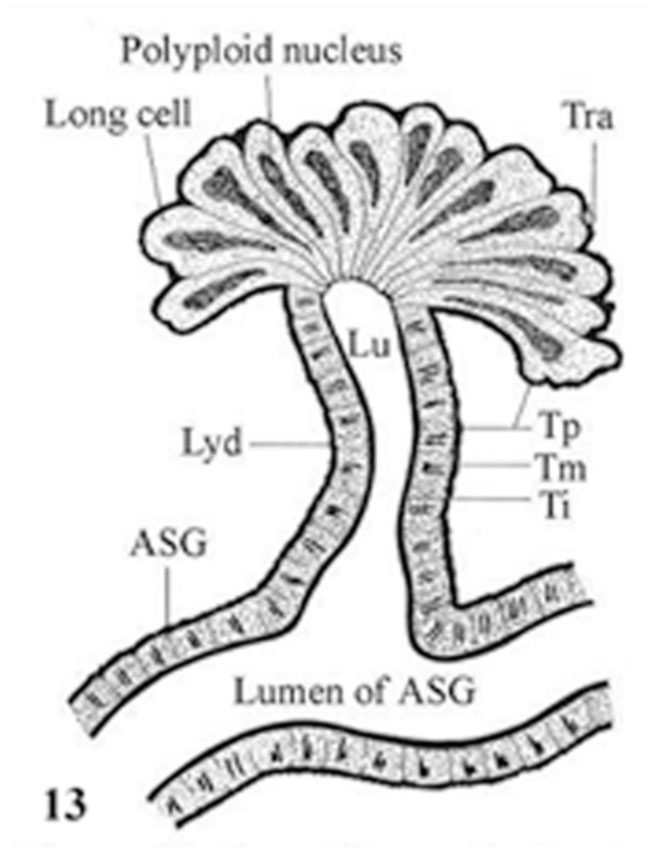
L. S. Lyonet's gland of *Antheraea mylitta* (diagrammatic) to show the continuity of different layers of its duct with that of the anterior silk gland. High quality figures are available online.

The basic histological features of Lyonet's gland in the second to fifth instar larvae were similar (Figure 7). Each gland was composed of long cells of various lengths, arranged in whorls. The longest cell measured had a length of ∼140µ. The whorls of long cells were wrapped by an extremely fine basal lamina for which the gland had a superficial rosette appearance. The bases of the glandular cells remained attached to the cuticular intimai layer of the Lyonet's gland duct (Figures 8, 9). Each glandular cell contained a long polyploid nucleus. These cells were found to be richly supplied with tracheoles. Fine nerve fibers were also found, ending over the surfaces of the gland.

The histology of the duct of Lyonet's gland was quite similar to that of ASG, the walls comprised of three layers: the outer most thin tunica propria, or basal lamina; a middle epithelial layer, or tunica media, made up of single layered non secretory low columnar cells with chromatin lumps; and the inner most thick tunica intima, or cuticular layer, surrounding the lumen of the duct. In fifth instar larvae, the thicknesses of tunica propria, tunica media, and tunica intima were 3 µ, 16µ and 5µ respectively. The lumen of the Lyonet's gland duct was 8µ wide.

The Lyonet's glands became fully functional in the late fifth instar. This is evidenced by the presence of few secretory granules in the Lyonet's gland cells of the fourth instar larvae, while in fifth instar larvae the glandular cells contained a much larger number of secretory granules.

**Figure 10.**
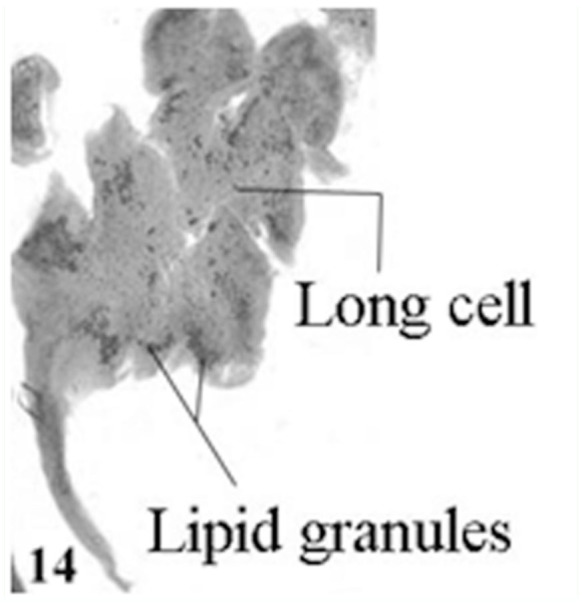
Lipid granules in Lyonet's gland cells of *Antheraea mylitta* (Sudan black B with neutral red as counter stain). High quality figures are available online.

The chemical nature of secretory materials of the Lyonet's gland cells was studied using histochemical stains. It was found that the Lyonet's gland cells were intensely mercuric bromophenol blue positive, indicating high protein content in the cells. Intense Periodic acid Schiff (PAS) reaction was confined to the basal and apical cytoplasm of the glandular cells, indicating high glycogen content in these regions. In the case of the Sudan Black-B reaction, the cells showed negative results, except for the clustered granules, which were intensely positive, indicating high lipid content in them (Figure 10).

## Discussion

The paired Lyonet's glands, characteristic of silk synthesizing Lepidoptera, have been studied in *Bombyx mori* (Waku and Sumimoto 1974), *Ostrinia nubilalis* (Drecktrah et.al. 1966), *Spodoptera frugiperda* (Chi et.al. 1975), and *Diatraea saccharalis* (Victoriano and Gregorio 2004). The results of the present study on the Lyonet's glands of *A. mylitta* revealed a similarity in the location and basic morphology of these accessory glands in all the species studied. However, as far as detailed morphology of these glands is concerned, in each case the arrangement of long glandular cells over the duct was different and characteristic for the species. It is also revealed that the basic histology of the gland and its duct were also similar.

The Lyonet's glands in *A*.*mylitta* appeared to be neuro-controlled, as they were supplied by fine nerves from the subesophageal ganglion. This was quite similar to that described in *D. saccharalis* (Victoriano and Gregorio 2004).

In *A. mylitta*, the histology of the Lyonet's gland duct was exactly the same as that of the ASG (Patra 2008). Furthermore, there is a clear continuity of the three layers (tunica propria, tunica media, and tunica intima), as well as the lumina of ASG, and the Lyonet's gland duct. This indicates that the Lyonet's gland duct was formed as a result of the out-pushing of the walls of the ASG during early development. The origin of the glandular cells of Lyonet's gland appeared to be due to the enormous elongation of tunica media cells of the duct at the growing end. These assumptions, however, require a detailed study of the embryonic development of the Lyonet's gland and its duct.

Although the role played by the Lyonet's gland is still not clear, histochemical studies of the gland cells in *A. mylitta* have clearly revealed the presence of clustered lipid granules in their cytoplasm, which may be secreted as a lubricating substance, facilitating the extrusion of silk from the silk glands. A similar suggestion has been made by Glasgow (1936) and Day and Waterhouse (1953).

## Abbreviations

ASG,: anterior part of the silk gland;
Cd,: common silk gland duct;
Hyp,: hypopharynx;
LLy: left Lyonet's gland;
Lu,: lumen;
Lyd,: Lyonet's gland duct;
Msp,: muscles of silk press;
Rly: right Lyonet's gland;
Sp,: silk press;
Spn,: spinneret;
Ti,: tunica intima;
Tm,: tunica media;
Tp,: tunica propria;
Tra,: tracheoles
